# Ten-year clinical outcomes in N0 ER+ breast cancer patients with Recurrence Score-guided therapy

**DOI:** 10.1038/s41523-019-0137-3

**Published:** 2019-11-08

**Authors:** Salomon M. Stemmer, Mariana Steiner, Shulamith Rizel, Noa Ben-Baruch, Beatrice Uziely, Debbie M. Jakubowski, Julie Baron, Steven Shak, Lior Soussan-Gutman, Avital Bareket-Samish, Georgeta Fried, Ora Rosengarten, Amit Itay, Bella Nisenbaum, Daniela Katz, Michelle Leviov, Margarita Tokar, Nicky Liebermann, David B. Geffen

**Affiliations:** 1 Davidoff Center, Rabin Medical Center, Petah Tikva, Israel; 2 Sackler Faculty of Medicine, Tel Aviv University, Tel Aviv, Israel; 3 Oncology Dept., Lin Medical Center, Haifa, Israel; 4 Oncology Dept., Kaplan Medical Center, Rehovot, Israel; 5 Sharett Institute of Oncology, Hadassah-Hebrew University Medical Center, Jerusalem, Israel; 6 Genomic Health Inc, Redwood City, CA USA; 7 Oncotest Division, Teva Pharmaceutical Industries, Ltd, Shoham, Israel; 8 BioInsight Ltd, Zichron Yaakov, Israel; 9 Oncology Dept., Rambam Health Care Campus, Haifa, Israel; 10 Oncology Institute, Shaare Zedek Medical Center, Jerusalem, Israel; 11 Oncology Dept., Sheba Medical Center at Tel HaShomer, Ramat Gan, Israel; 12 Oncology Dept., Meir Medical Center, Kfar Saba, Israel; 13 Oncology Dept., Assaf Harofeh Medical Center, Zerifin, Israel; 14 Department of Oncology, Soroka University Medical Center, Beer Sheva, Israel; 15 Faculty of Health Sciences, Ben-Gurion University of the Negev, Beer Sheva, Israel; 16 Community Division, Clalit Health Services, Tel Aviv, Israel

**Keywords:** Cancer therapy, Breast cancer

## Abstract

The 21-gene Recurrence Score (RS) assay is a validated prognosticator/predictor of chemotherapy (CT) benefit in early-stage estrogen receptor (ER)-positive breast cancer (BC). Long-term data from real-life clinical practice where treatment was guided by the RS result are lacking. We performed exploratory analysis of the Clalit Health Services (CHS) registry, which included all CHS patients with node-negative ER+ HER2-negative BC who underwent RS testing between 1/2006 and 12/2009 to determine 10-year Kaplan–Meier estimates for distant recurrence/BC-specific mortality (BCSM) in this cohort. The analysis included 1365 patients. Distribution of RS results: RS 0–10, 17.8%; RS 11–25, 62.5%; RS 26–100, 19.7%. Corresponding CT use: 0, 9.4, and 69.9%. Ten-year distant recurrence rates in patients with RS 0–10, 11–25, and 26–100: 2.6% (95% confidence interval [CI], 1.1–6.2%), 6.1% (95% CI, 4.4–8.6%), and 13.1% (95% CI, 9.4–18.3%), respectively (*P* < 0.001); corresponding BCSM rates: 0.7% (95% CI 0.1–5.1%), 2.2% (95% CI, 1.3–3.7%), and 9.5% (95% CI, 6.0–14.9%) (*P* < 0.001). When the analysis included patients treated with endocrine therapy alone (95.5/87.5% of patients with RS 0–10/11–25), 10-year distant recurrence and BCSM rates for RS 0–10 patients were 2.7% (95% CI, 1.1–6.5%) and 0.8% (95% CI, 0.1–5.3%), respectively, and for RS 11–25 patients, 5.7% (95% CI, 3.9–8.3%) and 2.0% (95% CI, 1.1–3.7%), respectively. For RS 11–25 patients, no statistically significant differences were observed in 10-year distant recurrence/BCSM rates between CT-treated and untreated patients; however, this should be interpreted cautiously since the number of events was low and patients were not randomized. In conclusion, in node-negative ER+ HER2-negative BC patients, where treatment decisions in real-life clinical practice incorporated the RS, patients with RS 0–25 (~80% of patients, <10% CT use) had excellent outcomes at 10 years. Patients with RS 26–100 had high distant recurrence risk despite CT use and are candidates for new treatment approaches.

## Introduction

The 21-gene Oncotype DX Breast Recurrence Score® assay is a validated prognostic/predictive tool used to guide adjuvant treatment decisions in estrogen receptor (ER)+ HER2-negative early breast cancer (BC).^1–7^ The initial validation used a prospective–retrospective design. Since then, the assay has been further validated with data from two phase 3 trials (TAILORx, WSG-PlanB), as well as with analyses of the SEER registry and the National Cancer Data Base.^4–8^

Clalit Health Services (CHS), the largest HMO in Israel, started reimbursing the assay in 2006. We have previously reported an analysis of the CHS registry focusing on treatment decisions/clinical outcomes in RS-tested node-negative BC patients whose treatment decisions in clinical practice incorporated the RS results.^9^ Our analyses of 1801 patients with ~6 years of median follow-up demonstrated that 5-year clinical outcomes (risk of distant recurrence, breast cancer-specific mortality [BCSM]) were consistent with the RS validation studies further confirming its clinical utility. Specifically, our findings supported the use of endocrine therapy (ET) alone in node-negative patients with RS 0–25.^9^

It is well established that ER+ BC patients have a protracted recurrence risk with approximately half of all distant recurrences occurring after 5 years and a continuum of relapse until 20 years.^10^ Thus far, the only registry reporting long-term clinical outcomes by RS and chemotherapy (CT) use was the SEER registry; however, they reported 9-year BC-specific survival (BCSS) findings, and do not collect distant recurrence events.^4^ Our goal in this analysis of the maturing data from the CHS registry was to investigate 10-year distant recurrence/BCSM rates by using the TAILORx^6^ RS categorization.

## Results

### Patient characteristics

This exploratory analysis (*N* = 1365) includes a subset of the 1801 patients from the original analysis^9^ for whom long-term outcome data were available. Patient characteristics in this subset were similar to those in the original analysis. The median follow-up of the current analysis was 9.0 years (interquartile range: 5.9–10.0 years). Patients were mostly females (99.3%), median age was 60 (interquartile range, 52–66) years, 50.3% had grade 2 tumors, 55.3% had tumors >1–2 cm, and 80.8% had invasive ductal carcinoma (Table 1).

**Table 1:** Baseline patient and tumor characteristics

	*N* = 1365
Female, *n* (%)	1355 (99.3%)
Median (interquartile range) age, years	60 (52–66)
Age category, %	
<40 years	33 (2.4%)
40–49 years	183 (13.4%)
50–59 years	458 (33.6%)
60–69 years	473 (34.7%)
70–79 years	201 (14.7%)
≥80 years	17 (1.3%)
Median (interquartile range) tumor size in the greatest dimension, cm	1.5 (1.1–2.0)
Tumor size category, *n* (%)	
≤1 cm	297 (21.8%)
>1–2 cm	755 (55.3%)
>2	303 (22.2%)
Unknown	10 (0.7%)
Tumor-grade category, *n* (%)	
Grade 1	196 (14.4%)
Grade 2	687 (50.3%)
Grade 3	221 (16.2%)
Not applicable/unknown^a^	261 (19.1%)
Histology, *n* (%)	
IDC	1103 (80.8%)
ILC	160 (11.7%)
Papillary	15 (1.1%)
Mucinous/colloid	40 (2.9%)
Other/unknown	47 (3.4%)

*IDC* invasive ductal carcinoma, *ILC* invasive lobular carcinoma

^a^Fifty-eight percent of unknown tumor grade are ILC

### RS distribution and adjuvant CT use

Overall, 243 patients (17.8%) had RS 0–10, 853 (62.5%) had RS 11–25, and 269 (19.7%) had RS 26–100. The RS 11–25 group was subdivided into three subgroups (RS 11–15, *n* = 273, 20.0% of all patients; RS 16–20, *n* = 344, 25.2% of all patients; RS 21–25 *n* = 236, 17.3% of all patients). CT use was 0% in RS 0–10, 9.4% in RS 11–25, and 69.9% in RS 26–100 patients. Within the RS 11–25 group, CT use was 1.8, 7.0, and 21.6% in RS 11–15, 16–20, and 21–25 patients, respectively.

### Distant recurrence and BCSM rates

**Fig. 1:**
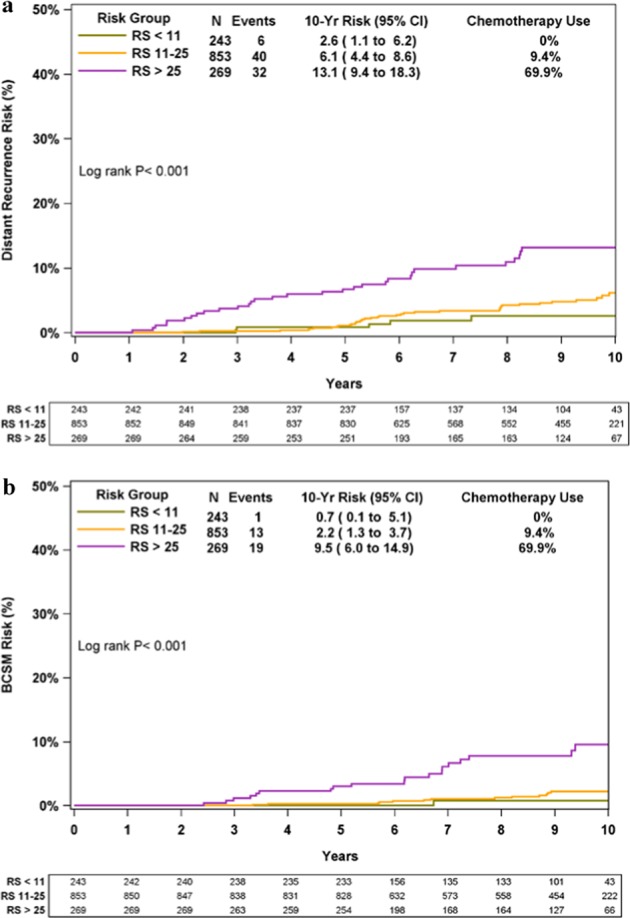
KM distant recurrence and BCSM curves by RS risk groups. The box under each graph presents the number of patients at risk at each time point. Two-degree of freedom log-rank *P*-values were calculated from all the data. BCSM breast cancer-specific mortality

KM estimates for 10-year distant recurrence/BCSM rates differed significantly between the RS groups (*P* < 0.001 for both outcomes). Ten-year Kaplan–Meier (KM) distant recurrence rates in the RS 0–10, 11–25, and 26–100 groups were 2.6% (95% confidence intervals [CI], 1.1–6.2%), 6.1% (95% CI, 4.4–8.6%), and 13.1% (95% CI, 9.4–18.3%), respectively (Fig. 1a). Ten-year KM BCSM rates in these respective groups were 0.7% (95% CI, 0.1–5.1%), 2.2% (95% CI, 1.3–3.7%), and 9.5% (95% CI, 6.0–14.9%) (Fig. 1b).

An analysis of distant recurrence risk by RS groups within subgroups defined by age/tumor size/tumor grade revealed that the RS result continued to discriminate between low- and high-risk disease (Supplementary Fig. 1).

### Distant recurrences and BCSM in years 0–5 and >5–10

**Fig. 2:**
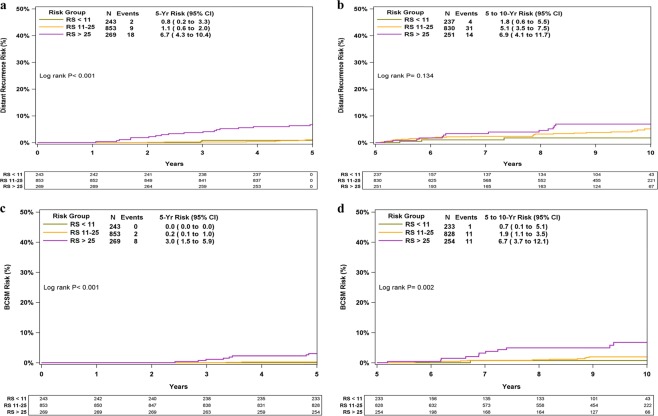
KM distant recurrence and BCSM curves for years 0–5 and >5–10 by RS group. The box under each graph presents the number of patients at risk at each time point. Two-degree of freedom log-rank *P*-values were calculated from all the data. BCSM breast cancer- specific mortality

Distant recurrence/BCSM rates were evaluated for years 0–5 and >5–10 separately (Fig. 2). All RS groups demonstrated protracted risk of recurrence/BCSM death with 49/78 recurrences and 23/33 BC deaths occurring after 5 years. The difference in KM estimates for distant recurrence and BCSM between the RS groups was statistically significant in years 0–5 (*P* < 0.001), whereas for years >5–10, the difference was significant only for BCSM (*P* = 0.002).

### Risk of distant recurrence/BCSM in patients treated with ET alone

**Fig. 3:**
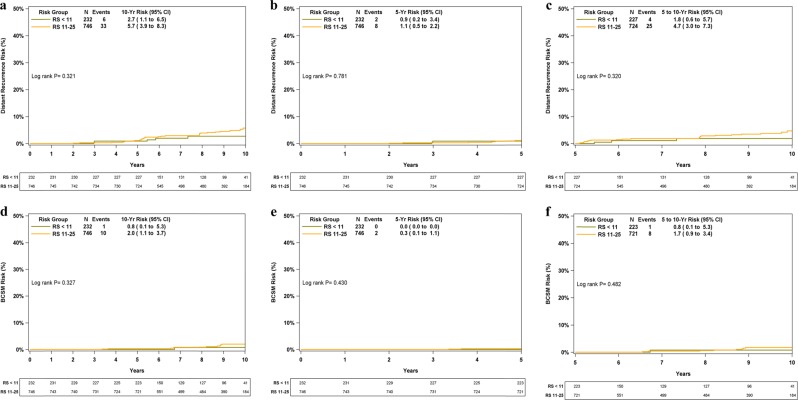
KM distant recurrence and BCSM curves in patients with RS 0–10 and 11–25 who were treated with ET alone for the entire 10 years, for years 0–5, and >5–10, separately. The box under each graph presents the number of patients at risk at each time point. One-degree of freedom log-rank *P-*values were calculated from all the data. BCSM breast cancer-specific mortality

The assay was developed to predict prognosis and CT benefit in patients receiving ET. In addition to not receiving CT, a small subset of patients (4%) did not receive ET for various reasons. Therefore, we performed an analysis on the 95.5% of RS 0–10 patients and the 87.5% of RS 11–25 patients who received ET alone (Fig. 3). The KM 10-year distant recurrence rates in these patients were 2.7% (95% CI, 1.1–6.5%) in RS 0–10 patients and 5.7% (95% CI, 3.9–8.3%) in RS 11–25 patients (Fig. 3a). The corresponding KM 10-year BCSM rates were 0.8% (95% CI, 0.1–5.3%) and 2.0% (95% CI, 1.1–3.7%) (Fig. 3d). Similar to the analysis performed for all patients by the RS group, an analysis focused on patients with ET alone and RS 0–25, for years 0–5 and >5–10 separately demonstrated protracted risk for distant recurrence/BC death with 29/39 of distant recurrences and 9/11 of BC deaths occurring after 5 years (Fig. 3b, c, e, f).

### Distant recurrence risk/BCSM by RS, clinical risk, and adjuvant CT use

**Fig. 4:**
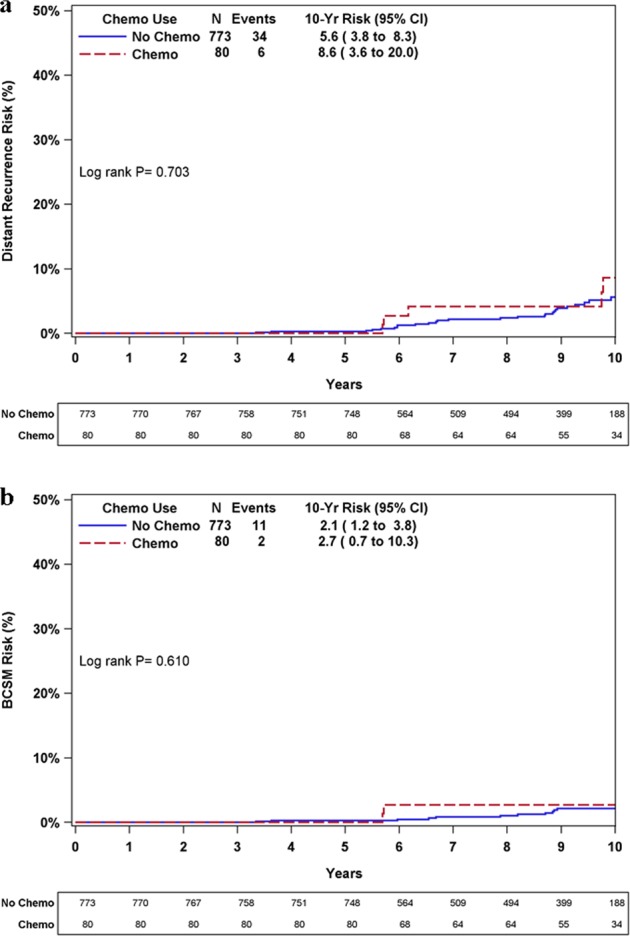
KM distant recurrence and BCSM curves in patients with RS 11–25 by adjuvant CT use. The box under each graph presents the number of patients at risk at each time point. One-degree of freedom log-rank *P*-values were calculated from all the data. BCSM breast cancer-specific mortality

Analysis of distant recurrence risk/BCSM by adjuvant CT use for RS 11–25 patients revealed that KM estimates for 10-year distant recurrence/BCSM rates were not statistically significantly different between CT-treated and untreated patients (*P* = 0.703 and *P* = 0.610, respectively) (Fig. 4). This analysis was not performed for RS 0–10 patients as none of them received adjuvant CT, or for RS 26–100 patients as only ~30% of them did not receive adjuvant CT. Subdividing the RS 11–25 group (*n* = 853) revealed that in this RS category, 9/273, 13/344, and 16/236 distant recurrence events were observed in RS 11–15, 16–20, and 21–25 patients, respectively. Exploratory analyses in RS 11–25 patients by clinical risk and adjuvant CT use showed that KM estimates for 10-year distant recurrence/BCSM rates were not statistically significantly different between CT-treated and untreated patients, irrespective of clinical risk (Supplementary Fig. 2).

Notably, in these analyses, study samples and the number of events (particularly when stratifying by clinical risk) were small, and patients were not randomized and therefore prone to selection bias. The sample size and design of this study limits its ability to identify possible true differences in outcomes between CT-treated and untreated patients.

### Multivariable analysis

Multivariable regression analysis was performed on 1098 patients (267 were excluded due to missing data, predominantly tumor grade). The model of distant recurrence risk for years 0–10 included the RS group (11–25 vs 0–10; 26–100 vs 0–10), age (50–69 vs <50 years; ≥70 vs <50 years), tumor size (≥2 vs <2 cm), and grade (2 vs 1; 3 vs 1). RS group, age, and tumor size were all significantly associated with distant recurrence risk, whereas grade was not (Supplementary Table 1). The hazard ratio (HR) for RS 11–25 vs 0–10 was 2.15 (95% CI, 0.75–6.21) and for RS 26–100 vs 0–10, 4.91 (95% CI, 1.63–14.82) (*P* = 0.002). The HR for 50–69 vs <50 years was 0.93 (95% CI, 0.44–1.95) and for ≥70 vs <50 years, 2.42 (95% CI, 1.05–5.55) (*P* = 0.007). The HR for ≥2 vs <2 cm was 2.72 (95% CI, 1.60–4.63) (*P* < 0.001).

## Discussion

These are the first reported 10-year outcome data including distant recurrence risk from a large cohort of unselected patients where the RS was included in adjuvant treatment decisions in real-life clinical practice. As previously shown,^9^ adjuvant CT use was consistent with the RS. Long-term clinical outcomes were very good in RS 0–25 patients who received ET alone (no RS 0–10 patient received CT, and in RS 11–25 patients, there was no statistically significant difference in distant recurrence risk between CT-treated and untreated patients). Our results are very consistent with all recent studies in which similar cutoff values were used, including TAILORx, WSG-PlanB, and reanalysis of the NSABP B-20 trial, as well as recent analyses of large datasets from the United States including the recent analyses of the SEER registry and the National Cancer Data Base.^4–8^

We demonstrated the clinical utility of the RS for predicting risk of distant recurrence in years 0–5 and in years 0–10. Five-year outcome data are most informative for CT treatment decisions as the benefit from CT is primarily obtained in the first 2–3 years after treatment. The prognostic ability of the RS with respect to 10-year risk of distant recurrence is important as patients with ER+ HER2-negative disease recur at a steady rate from 5 to 20 years.^10^ We observed that for all patients and particularly those with RS 0–25, recurrence risk from year 5 to 10 was substantial and numerically higher than that in the first 5 years. Notably, the analysis of the NSABP B-28, in which patients were treated with CT followed by 5 years of tamoxifen showed that the RS was prognostic in years 0–5 and beyond year 5 (for the entire cohort).^11^ In our cohort, the RS was prognostic (with respect to distant recurrence) for years 0–5 and 0–10 but not for years >5–10. Interestingly, in NSABP B-14, analysis of all patients (in the tamoxifen-only arm) demonstrated that the RS was not prognostic beyond year 5. However, stratifying the B-14 patients according to the expression of the estrogen receptor gene (*ESR1*) from the RS result revealed that in patients with high *ESR1* expression, the RS was prognostic beyond year 5, whereas in patients with low *ESR1* expression it was not.^11^ Analyses exploring the expression of *ESR1* and the duration of ET received in our cohort are ongoing.

In our exploratory analysis of clinical outcomes in CT-treated and untreated patients with RS 11–25, no CT benefit was observed irrespective of clinical risk (as determined by grade and tumor size). These results are consistent with the very recent TAILORx exploratory analysis focusing on 9427 patients for whom the same clinical risk assessment was made.^12^ Notably, the TAILORx analysis stratified patients further, by age (≤50, >50 years) and by RS subgroups (RS 11–15, 16–20, and 21–25), and found trends suggesting CT benefit in patients ≤50 years and RS 21–25; however, these trends did not vary by clinical risk.^12^ Further stratification in our study was prohibited by the smaller sample size and the small number of events in our cohort.

Our multivariable analysis demonstrated that the RS group, age, and tumor size were independent predictors of distant recurrence, whereas grade was not. This finding regarding grade may be explained by the reported significant interobserver variability in the assessment of histologic grade.^9,13,14^ Importantly, patients with RS 0–25 had excellent outcomes, including those with grade 3 tumors. Interestingly, in earlier multivariable analyses (including ours),^1,2,9^ age was not significantly associated with distant recurrence, whereas in the current analysis, patients ≥70 years had significantly higher distant recurrence risk compared with patients <50 years (HR, 2.42; 95% CI, 1.05–5.55; *P* = 0.007). The higher risk in the elderly population may stem from undertreatment with CT and other treatment modalities in this population (CT use in patients ≥70 years, 0, 3.6, and 38.1% in patients with RS 0–10, 11–25, and 26–100, respectively vs 0, 17.5, and 82.0%, respectively, in patients <50 years), a common phenomenon shown in multiple studies worldwide.^15^ Interestingly, TAILORx also observed some interesting findings with regard to patient age. There, an exploratory analysis of the randomized arms (RS 11–25) demonstrated some CT benefit in younger (≤50 years) patients with RS 16–25 (although overall in these arms, and for those >50 years, ET was not inferior to CT). This observation may be, at least partly, explained by CT-induced menopause in younger patients.^6^ Our study design and small sample size prohibited us from exploring the potential interaction between age and the effect of CT in our cohort.

Our analysis adds to the available evidence on the utility of the RS, as unlike randomized trials such as TAILORx,^6,12^ our analysis reflects real-life clinical practice on a national level in which all patients regardless of age, gender, location, socioeconomic status, and comorbidities were included, and unlike the SEER registry analysis,^4^ where BCSM was the only clinical outcome captured, our study included analyses of BCSM as well as of distant recurrence. Notably, the analysis of the National Cancer Data Base focused on patients with RS 11–30 and the only outcome measure captured was overall survival.^8^ Our study is limited though, by its nonrandomized design, the potential selection bias with respect to patients being tested, the small sample size, and the small number of events in certain analyses. In addition, certain variables were not captured/included in the analysis such as CT regimens, type/duration of ET, and the rate of ovarian suppression. Last, though the details for each patient were unavailable, we do know that patients were not treated uniformly with respect to CT/ET regimens (e.g., tamoxifen vs aromatase inhibitors and duration of ET).

In conclusion, our results are remarkably consistent with those from TAILORx. Our findings demonstrate excellent outcomes for patients with RS 0–10, and suggest that for the majority of node-negative ER+ HER2-negative BC patients with RS 11–25, adjuvant CT may be safely spared. In contrast, patients with RS 26–100 had poorer outcomes despite high CT use and should be candidates for new treatment approaches.

## Methods

### Study design and patient population

This retrospective analysis of the prospectively designed CHS registry investigated 10-year distant recurrence/BCSM rates in patients with node-negative ER+ HER2-negative BC in clinical practice. It is an exploratory analysis of a subset of patients from the registry analysis previously reported for whom long-term outcome data are available.^9^ Collecting outcome data from all CHS patients undergoing the 21-gene assay was planned by CHS, in concert with assay reimbursement approval. This analysis includes all CHS patients with node-negative ER+ HER2-negative BC who underwent 21-gene testing between 1/2006 (CHS approval of the assay) and 12/2009. Exclusion criteria were as previously described.^9^ In brief, key exclusion criteria included treatment with adjuvant trastuzumab, neoadjuvant treatment, metastatic disease at the time of testing/within 6 months of testing, and receiving adjuvant therapy for another malignancy within 6 months of testing.

The study was approved by the institutional review boards of the CHS Community Division and participating medical centers, and was granted a waiver for obtaining patient consent. The study was conducted in accordance with the Declaration of Helsinki.

### Data source

The following data sources were used: The Teva Pharmaceutical Industries database (for RS results and patient/tumor characteristics), medical records, and CHS claims information (for treatments received and recurrence/death). Those extracting the clinical information were unaware of the RS result.

### Statistical analysis

The log-rank test was used to compare 10-year KM estimates for distant recurrence rates (primary endpoint) and 10-year BCSM (secondary endpoint) between RS groups. HR and 95% CI values were calculated by using Cox regression models to evaluate the association of the RS group (11–25 vs 0–10, 26–100 vs 0–10), age (50–69 vs <50, ≥70 vs <50 years), tumor size (≥2 vs < 2 cm), and grade (2 vs 1, 3 vs 1) with distant recurrence. Clinical risk was assessed as in the recent TAILORx analysis.^12^ It was defined as low in patients with grade 1 and tumor diameter ≤3 cm, grade 2 and tumor diameter ≤2 cm, or grade 3 and tumor diameter ≤1 cm, and as high for all other cases. Patients with missing grade information were excluded from the analysis. Patients were censored at the time of the last follow-up, date of medical records review, or time of death (due to any cause). BC deaths were defined as those where patients had metastatic disease at the time of death. SAS 9.4 (SAS Institute Inc., Cary, NC) was used for the analysis. *P* < 0.05 was considered statistically significant and all tests were two-sided.

### Reporting summary

Further information on research design is available in the Nature Research Reporting Summary linked to this article.

## Supplementary information

Supplemental information

Reporting Summary Checklist

## Data Availability

The data generated and analyzed during this study are described in the following data record: 10.6084/m9.figshare.9896591.^16^ The dataset supporting all the figures, tables, and supplementary files in the published article, is not publicly available in order to protect patient privacy, but can be accessed from the corresponding author on request, upon the completion of a Data Usage Agreement, as described in the data record above.

## Code availability

The codes used during the current study are available from the corresponding author on reasonable request without undue qualification.
