# Supplementary material for: Ten-year clinical outcomes in N0 ER+ breast cancer patients with Recurrence Score-guided therapy
Source: NPJ Breast Cancer. 2019 Nov 8;5:41. doi: 10.1038/s41523-019-0137-3 (PMC6841708; doi:10.1038/s41523-019-0137-3)
Supplement: Supplementary file 1 — Supplemental information [file 41523_2019_137_MOESM1_ESM.pdf]

SUPPLEMENTARY INFORMATION

Supplementary Figure 1. KM distant recurrence curves by RS groups and clinicopathological characteristics including age, tumor size, and tumor grade. For each RS category, the percentage of patients receiving chemotherapy is indicated. The box under each graph presents the number of patients at risk at each time point. Results for the subgroup analyses should be interpreted cautiously due to small number of patients in some of the subgroups, low event rates, and the potential for selection bias with respect to the patients being RS tested.

a

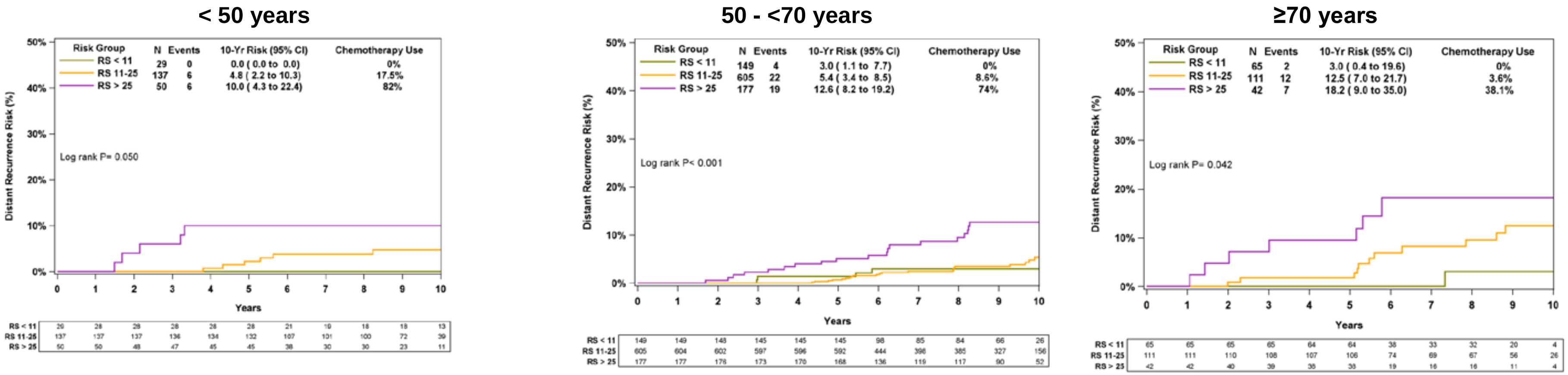

b

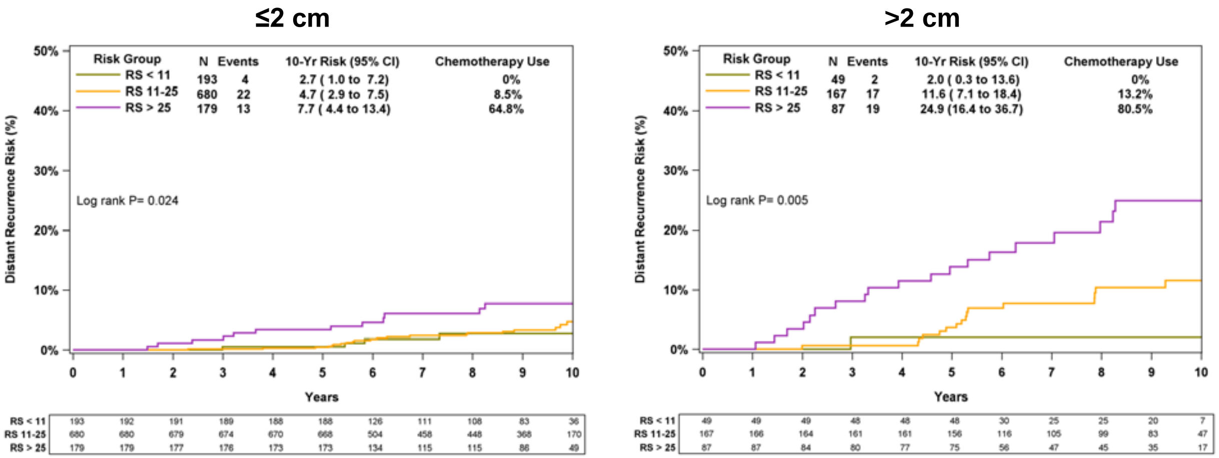

c

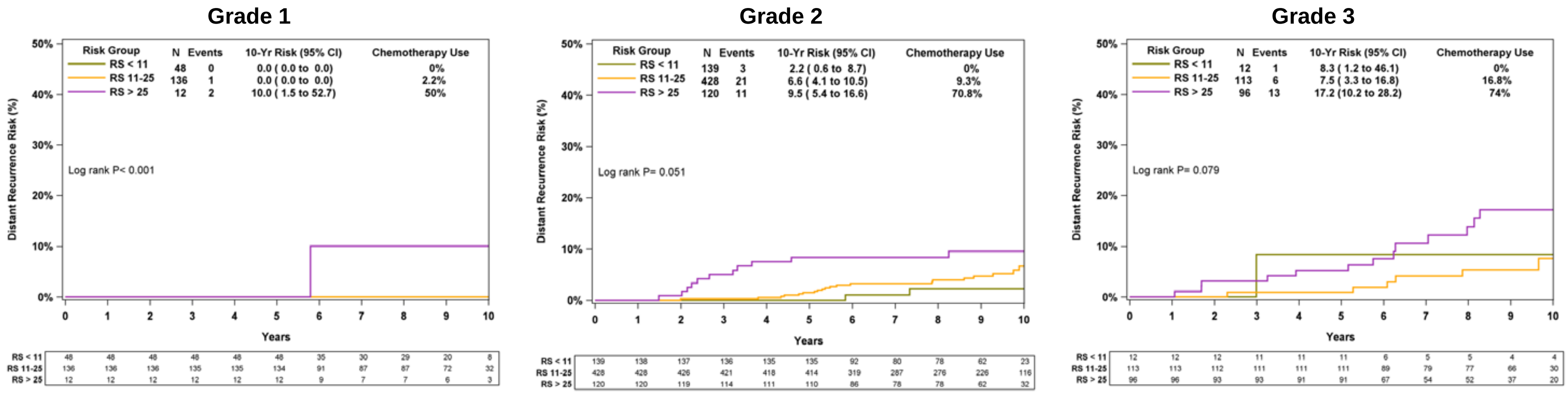

Grade 1
Risk Group | N | Events | 10-Yr Risk (95% CI) | Chemotherapy Use
RS < 11 | 48 | 0 | 0.0 (0.0 to 0.0) | 0%
RS 11-25 | 136 | 1 | 0.0 (0.0 to 0.0) | 2.2%
RS > 25 | 12 | 2 | 10.0 (1.5 to 52.7) | 50%
Log rank P< 0.001
Distant Recurrence Risk (%)
50% 40% 30% 20% 10% 0%
0 1 2 3 4 5 6 7 8 9 10
Years
RS < 11 48 48 48 48 48 48 35 30 29 20 8
RS 11-25 136 136 136 135 135 134 91 87 87 72 32
RS > 25 12 12 12 12 12 12 9 7 7 6 3
Grade 2
Risk Group | N | Events | 10-Yr Risk (95% CI) | Chemotherapy Use
RS < 11 | 139 | 3 | 2.2 (0.6 to 8.7) | 0%
RS 11-25 | 428 | 21 | 6.6 (4.1 to 10.5) | 9.3%
RS > 25 | 120 | 11 | 9.5 (5.4 to 16.6) | 70.8%
Log rank P= 0.051
Distant Recurrence Risk (%)
50% 40% 30% 20% 10% 0%
0 1 2 3 4 5 6 7 8 9 10
Years
RS < 11 139 138 137 136 135 135 92 80 78 62 23
RS 11-25 428 428 426 421 418 414 319 287 276 226 116
RS > 25 120 120 119 114 111 110 86 78 78 62 32
Grade 3
Risk Group | N | Events | 10-Yr Risk (95% CI) | Chemotherapy Use
RS < 11 | 12 | 1 | 8.3 (1.2 to 46.1) | 0%
RS 11-25 | 113 | 6 | 7.5 (3.3 to 16.8) | 16.8%
RS > 25 | 96 | 13 | 17.2 (10.2 to 28.2) | 74%
Log rank P= 0.079
Distant Recurrence Risk (%)
50% 40% 30% 20% 10% 0%
0 1 2 3 4 5 6 7 8 9 10
Years
RS < 11 12 12 12 11 11 11 6 5 5 4 4
RS 11-25 113 113 112 111 111 111 89 79 77 66 30
RS > 25 96 96 93 93 91 91 67 54 52 37 20

Supplementary Figure 2. KM distant recurrence and BCSM curves by adjuvant CT use in patients with RS 11-25, and low or high clinical risk. The box under each graph presents the number of patients at risk at each time point. One-degree of freedom log-rank *P*-values were calculated from all the data. BCSM, breast cancer specific mortality.

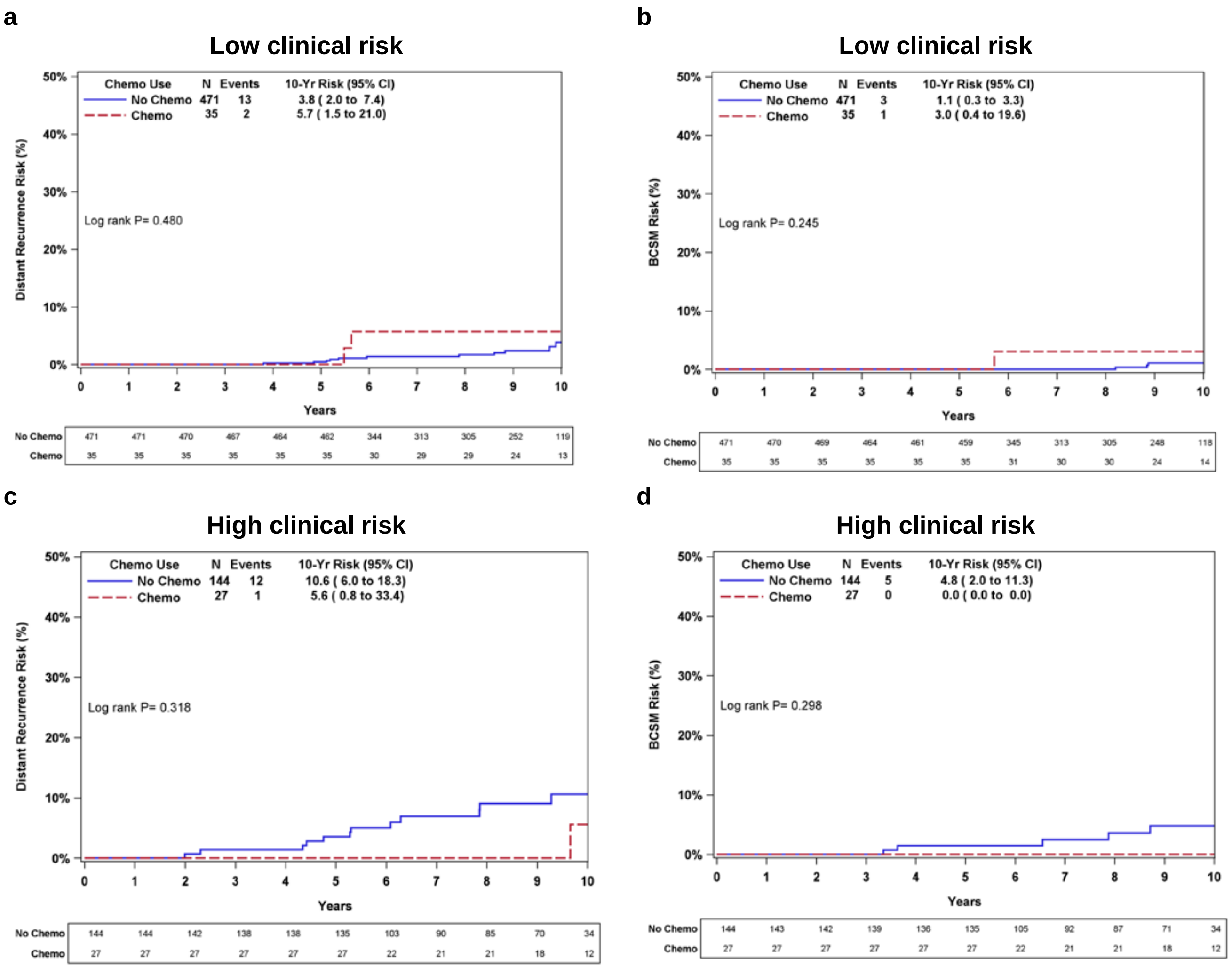

Supplementary Table 1. Multivariable model of 10-year risk of distant recurrence (*n* = 1098; 267 patients were excluded due to missing data).

Variable	Comparison	Hazard ratio (95% CI)	*P*-value
RS group	11-25 vs 0-10	2.15 (0.75-6.21)	0.002
	26-100 vs ≤0-10	4.91 (1.63-14.82)	
Age	50-69 vs <50	0.93 (0.44-1.95)	0.007
	≥70 vs <50	2.42 (1.05-5.55)	
Size	≥2 cm vs <2cm	2.72 (1.60-4.63)	<0.001
Grade	2 vs 1	2.57 (0.78-8.39)	0.272
	3 vs 1	2.75 (0.78-9.72)	
